# Population Pharmacokinetics of Colistin Sulfate in Critically Ill Patients: Exposure and Clinical Efficacy

**DOI:** 10.3389/fphar.2022.915958

**Published:** 2022-06-16

**Authors:** Xu-ben Yu, Xiao-Shan Zhang, Ye-Xuan Wang, Yu-Zhen Wang, Hong-Min Zhou, Fang-Min Xu, Jun-Hui Yu, Li-Wen Zhang, Ying Dai, Zi-Ye Zhou, Chun-Hong Zhang, Guan-Yang Lin, Jing-Ye Pan

**Affiliations:** ^1^ Department of Pharmacy, First Affiliated Hospital of Wenzhou Medical University, Wenzhou, China; ^2^ School of Pharmacy, Chonnam National University, Gwangju, South Korea; ^3^ School of Pharmacy, Wenzhou Medical University, Wenzhou, China; ^4^ Intensive Care Unit, First Affiliated Hospital of Wenzhou Medical University, Wenzhou, China; ^5^ Clinical Research Center, First Affiliated Hospital of Wenzhou Medical University, Wenzhou, China

**Keywords:** colistin sulfate, population pharmacokinetics, clinical efficacy, nephrotoxicity, dosing strategy

## Abstract

**Background:** Presently, colistin is commercially available in two different forms, namely, colistin sulfate and its sulphomethylated derivative, colistimethate sodium (CMS). However, in the currently reported studies, most of the clinical studies on colistin for parenteral use are referred to as CMS. Data on the pharmacokinetics (PK), clinical efficacy, and side effects of colistin sulfate in clinical use have not been reported.

**Methods:** This retrospective study was performed on carbapenem-resistant organism (CRO)-infected patients treated with colistin sulfate for more than 72 h. The population pharmacokinetic model was developed using the NONMEM program. The clinical outcomes including clinical treatment efficacy, microbiological eradication, and nephrotoxicity were assessed. Monte Carlo simulation was utilized to calculate the probability of target attainment (PTA) in patients with normal or decreased renal function.

**Results:** A total of 42 patients were enrolled, of which 25 (59.52%) patients were considered clinical treatment success and 29 (69.06%) patients had successful bacteria elimination at the end of treatment. Remarkably, no patient developed colistin sulfate-related nephrotoxicity. A total of 112 colistin concentrations with a range of 0.28–6.20 mg/L were included for PK modeling. The PK characteristic of colistin was well illustrated by a one-compartment model with linear elimination, and creatinine clearance (CrCL) was identified as a covariate on the clearance of colistin sulfate that significantly explained inter-individual variability. Monte Carlo simulations showed that the recommended dose regimen of colistin sulfate, according to the label sheet, of a daily dose of 1–1.5 million IU/day, given in 2–3 doses, could attain PTA > 90% for MICs ≤ 0.5 μg/mL, and that a daily dose of 1 million IU/day could pose a risk of subtherapeutic exposure for MIC ≥1 μg/ml in renal healthy patients.

**Conclusion:** Renal function significantly affects the clearance of colistin sulfate. A dose of 750,000 U every 12 h was recommended for pathogens with MIC ≤1 μg/ml. The dosage recommended by the label inserts had a risk of subtherapeutic exposure for pathogens with MIC ≥2 μg/ml. Despite higher exposure to colistin in patients with acute renal insufficiency, dose reduction was not recommended.

## Introduction

Polymyxins (colistin and polymyxin B) are old antimicrobials, which have been used clinically since the late 1960s, acting against Gram-negative bacteria such as *Acinetobacter baumannii, Pseudomonas aeruginosa, Klebsiella pneumoniae,* and *Escherichia coli* (Evans et al., 1999; Mohapatra et al., 2021). However, the clinical use of polymyxins has been restricted due to their side effects, such as neurotoxicity and nephrotoxicity. Recently, along with the emergence of multidrug-resistant (MDR) Gram-negative bacteria, polymyxins have been used as a first-line option for the treatment of carbapenem-resistant organism (CRO) infections (Tsuji et al., 2019).

Colistin is commercially available in two different forms, namely, colistin sulfate and colistimethate sodium (CMS) (Tsuji et al., 2019). CMS is an inactive prodrug of colistin and undergoes conversion into the active form to exert its bactericidal effect (Bergen et al., 2006). Unlike CMS, colistin sulfate is administered in its active form, whose pharmacokinetics is simpler than that of CMS. Therefore, it is important not to use the terms colistin and CMS interchangeably, as the chemistry, antibacterial activity, toxicity, and pharmacokinetics of these two entities differ substantially. Unfortunately, in the currently reported studies, it is not always possible to ascertain whether the “colistin” administered was colistin sulfate or CMS, albeit most of the clinical studies on colistin for parenteral use are referred to as CMS. It is because, in most countries, CMS is the only available form of colistin for parenteral use. However, there is a product of colistin sulfate for parenteral use which is available only in China (Chen et al., 2013). This parenteral product of colistin sulfate is labeled as 500,000 IU per vial (1 mg of pure colistin base = 17,000 IU of colistin) (Infectious Diseases Society of China, 2021). The recommended dosage regimen for intravenous use is 1–1.5 million IU per day, in 2–3 doses, in accordance with the manufacturer’s instructions. There is very limited pharmacological information in the literature on colistin sulfate in patients. Authors to this day still occasionally report and discuss “colistin” in generic terms which makes it hard to distinguish the preparation used (colistin sulfate or CMS). To the best of our knowledge, data on the pharmacokinetics (PK), clinical efficacy, and side effects of colistin sulfate in clinical use have not been reported. Previously, we reported the first case in which colistin sulfate was used for the treatment of MDR-Acinetobacter baumannii-induced post-neurosurgical ventriculitis in a 66-year-old male patient (Yu et al., 2022).

In the current study, we first developed a population PK model of colistin sulfate in critically ill patients, to identify the PK characteristics of colistin sulfate administered intravenously. Meanwhile, we analyzed the efficacy and side effects of colistin sulfate in the treatment of CRO infections. In addition, we performed Monte Carlo simulations to identify the probability of the pharmacokinetic/pharmacodynamics (PK/PD) target attainment of colistin sulfate with the dose regimens recommended according to the label sheet.

## Materials and Methods

### Patients and Ethics

This retrospective, observational study was designed in accordance with the Declaration of Helsinki and was approved by the Ethical Committees of the First Affiliated Hospital of Wenzhou Medical University, China ([2022]036). Adult patients receiving colistin sulfate for confirmed multi-resistant Gram-negative bacterial infections from January 2020 to December 2021 at the First Affiliated Hospital of Wenzhou Medical University were selected. The inclusion criteria were as follows: a) ≥18 years of age; b) receiving colistin sulfate (Shanghai New Asia Pharmaceuticals, Shanghai, China) for at least 3 days; and c) at least one plasma concentration of colistin was collected. The exclusion criteria were as follows: a) died within 24 h after being treated with colistin sulfate; b) does not receive colistin sulfate intravenously; and c) plasma concentrations obtained during renal replacement therapy or extracorporeal membrane oxygenation therapy were excluded when performing population pharmacokinetic modeling.

### Clinical Data Collection

Clinical data, including basic demographic characteristics, diagnoses, acute physiology and chronic health evaluation (APACHE) II score, infection sites, pathogenic bacteria and their sensitivity, laboratory values, medication information of colistin sulfate, treatment duration, and adverse events, were collected based on medical records.

Acute kidney injury (AKI) during the treatment of colistin sulfate was assessed using RIFLE criteria. AKI was defined as a 1.5-fold or more increase in serum creatinine and/or a decrease in the glomerular filtration rate (GFR) of 25% or more (Han et al., 2022). The definition of nephrotoxicity caused by colistin sulfate was further confirmed using the Naranjo criteria.

### Colistin Sulfate Administration and Sample Collection

The decision to administer colistin sulfate and its dosing regimen (dose amount, dosing interval, ways of administration, and treatment duration) was made by the attending physician. A loading dose of 1 million IU and a daily dose of 1.5 million IU divided into 2–3 times (maintenance dose) administered using an intravenous drip was recommended by the label sheet of colistin sulfate. In addition, for patients with pulmonary infection, inhalation of colistin sulfate (250,000 IU q12 h) was combinedly used; for patients with intracranial infection, colistin sulfate (50,000 IU q24 h) administered by the intraventricular/intrathecal (IVT/IT) route was combinedly used.

Plasma samples of colistin sulfate were collected after reaching the steady-state (attained after at least six doses). Dates and exact time of colistin sulfate administration and plasma sample collection were able to be indexed from the medical records. Plasma samples were separated by centrifugation for 5 min at 15,000 rpm immediately after sampling. The quantification of plasma concentration of colistin sulfate was performed using a validated high-performance liquid chromatography-tandem mass spectrometry assay (Gobin et al., 2010). The calibration ranges for colistin were 0.1–20 μg/ml. The method validations including calibration curve, selectivity, accuracy, precision, matrix effect, recovery, and stability met the requirement of FDA principles.

### Population Pharmacokinetic Modeling of Colistin Sulfate

PopPK analysis was performed using the nonlinear mixed-effects modeling program NONMEM (version 7.4, Icon Development Solutions, Ellicott City, MD, USA) and Pirana (version 2.9.7). R (version 3.6.0) and Xpose (version 4.3.2) software packages were applied to generate diagnostic plots.

One- and two-compartment structural models with first-order elimination were explored for the concentration–time profiles of colistin sulfate. Between-subject variability (BSV) was assessed using an exponential function. Residual variability was assessed using additive, proportional, or combined (additive plus proportional) error models. The base model was selected based on the visual inspection of diagnostic plots and goodness-of-fit criteria, including precision and plausibility of parameter estimation and improvement of the objective function value (OFV).

Relationships between individual empirical Bayesian estimates of PK parameters and patient covariates were examined visually. Covariates were included using a stepwise forward selection process until no further decrease in OFV was observed. All of the significant covariates were then incorporated into the basic model to construct a full model. The included covariates were further assessed using backward elimination. The additional criterion for retaining the covariate in the final model was the decrease in the unexplained BSV and increase in PK parameter estimate precision.

Goodness-of-fit plots, nonparametric bootstrap, and visual predictive check were performed to evaluate the final model and parameter estimates. A nonparametric bootstrap procedure was conducted to assess the performance and stability of the final model. Random sampling with replacement was utilized to generate 1,000 replicate datasets using the individual as the sampling unit. The median and 95% confidence intervals of the resulting parameters were calculated and compared with the final parameter estimates obtained using the NONMEM program. To evaluate the predictive performance, the statistics of the observed and simulated time–concentration profiles were compared using prediction- and variability-corrected visual predictive check. The dataset was simulated 1,000 times using the $SIMULATION block in NONMEM for prediction- and variability-corrected visual predictive check. The 90% CI for the 5th, 50th, and 95th percentiles of the simulated concentrations were calculated, plotted against time after the last dose, and compared with the observed concentrations.

### Monte Carlo Simulations

Monte Carlo simulations were performed using the final model on 60,000 virtual patients to identify the pragmatic dose adjustment of colistin sulfate. The ratio of the unbound concentration–time curve to the MIC (fAUC/MIC) has been shown to be the PK/PD index that best predicts bacterial killing for colistin (Dudhani et al., 2010a; Dudhani et al., 2010b; Bergen et al., 2010). The fAUC/MIC value for 1-log bacterial killing was approximately 15 for colistin sulfate against *Pseudomonas aeruginosa* and *Acinetobacter baumannii* in the mice thigh infection model (Cheah et al., 2015), which was used as the target. The probability of target attainment (PTA) of fAUC/MIC was calculated for each maintenance dose (500 KU q12 h, 500KU q8 h, 750 KU q12 h, 750KU q8 h, 1MU q12 h, and 1MU q8 h), with a loading dose (2 × maintenance dose), combined with various MICs (0.5, 1, 2 mg/L). The unbound fraction was defined as 0.5 (Cheah et al., 2015). PTA >90% was the target for dose selection in our study.

## Results

### Baseline Characteristics of Patients

A total of 42 hospitalized adult patients with 112 plasma concentrations were included. The demographic data of the patients, including basic information, diagnostic information, and microbial information, are summarized in Table 1. Most of the patients were male elderly people, and 76.19% of the included patients received mechanical ventilation; 33.33% of patients had a multiple site infection, and 69.05% of patients had an infection of the respiratory tract. In addition, the highest proportion of isolated CRO was *Acinetobacter baumannii* (73.81%), followed by *Pseudomonas aeruginosa* (16.67%), *Klebsiella pneumoniae* (7.14%), and *Enterobacter cloacae* (2.24%).

**TABLE 1:** Clinical characteristics of patients.

Characteristic	Value^a^
Age (years)	67.90 ± 13.74
Sex (male/female)	37/5
Body weight (kg)	63.47 ± 9.64
Serum creatinine (μmol/L)	116.64 ± 105.49
Creatinine clearance (ml/min)^b^	79.54 ± 53.99
APACHE II score	17 [14, 26]
Mechanical ventilation	32 (76.19%)
Vasoactive agents	15 (35.71%)
Infection site	
Multiple	14 (33.33%)
Respiratory tract	29 (69.05%)
Blood	11 (26.19%)
Intracranial	4 (9.52%)
Abdomen	10 (23.81%)
Digestive tract	2 (7.14%)
Pathogenic bacteria	
*Acinetobacter baumannii*	31 (73.81%)
*Klebsiella pneumoniae*	3 (7.14%)
*Pseudomonas aeruginosa*	7 (16.67%)
*Enterobacter cloacae*	1 (2.24%)
MIC ≤0.5 μg/ml	14 (33.33%)
MIC = 1 μg/ml	21 (50%)
MIC ≥2 μg/ml	0 (0.00%)
Administration	
Intravenous drip	42 (100%)
Intrathecal injection	4 (10%)
Inhalation	22 (52.38%)
Treatment duration (days)	11.63 ± 5.96
Daily dose (IU)	150 [150, 200]
Combination	
Carbapenems	17 (52.38%)
Tigecycline	7 (16.67%)
Cefoperazone–sulbactam	20 (47.62%)
Ceftazidime–avibactam	1 (7.14%)

a Values are no. (%) or median [min, max] or mean ± SD.

b Creatinine clearance calculated using the Cockcroft–Gault equation.

### Medications and Outcomes

All patients had the treatment with colistin sulfate intravenously. Patients with pulmonary infection were combinedly treated with colistin sulfate (250,000 IU q12 h) administered by inhalation, and patients with intracranial infection were combinedly treated with colistin sulfate (50,000 IU q24 h) administered by the IVT/IT route. The treatment duration was 11.63 ± 5.96 days. In all patients, colistin sulfate was combinedly used with another antimicrobial for the treatment of isolated CRO. Carbapenems were the agents combined the most (52.38%), followed by cefoperazone–sulbactam (47.62%), tigecycline (16.67%), and ceftazidime–avibactam (7.14%).

A total of 25 (59.52%) patients were considered exhibiting clinical treatment success, which was defined as improvements of clinical symptoms and parameters including body temperature, biochemistry indicators of infection (white cell count in adult ≤109, C-reactive protein ≤10 mg/L, procalcitonin <0.05 ng/ml, and erythrocyte sedimentation rate <15 mm/h), and clinician-documented improvement at the end of treatment. The 30-day mortality rate was 23.81%, and the 14-day mortality rate was 16.67%; 69.05% of patients had successful bacteria elimination at the end of treatment. Remarkably, two patients developed AKI during the treatment of colistin sulfate. However, the AKIs were not considered as colistin’s nephrotoxicity according to the Naranjo criteria. The AKIs were considered caused by infections because the renal function of these two patients was improved (decreased creatinine levels and increased urine amount) after a few days of starting colistin treatment, accompanied by their improved clinical symptoms (Table 2).

**TABLE 2:** Total outcomes of patients.

Outcome	Value^a^
Colistin MIC ≥2 mg/L	0 (0.00%)
AUC (mg·h/L)	39.39 ± 14.47
14-day mortality rate	7 (16.67%)
30-day mortality rate	10 (23.81%)
Duration of hospitalization (days)	54.47 ± 27.59
Clinical anti-infective success	25 (59.52%)
Bacteria elimination (yes)	29 (69.05%)
Nephrotoxicity (yes)	0 (0.00%)

a Values are no. (%) or mean ± SD.

### Population Pharmacokinetic Analysis

**FIGURE 1:**
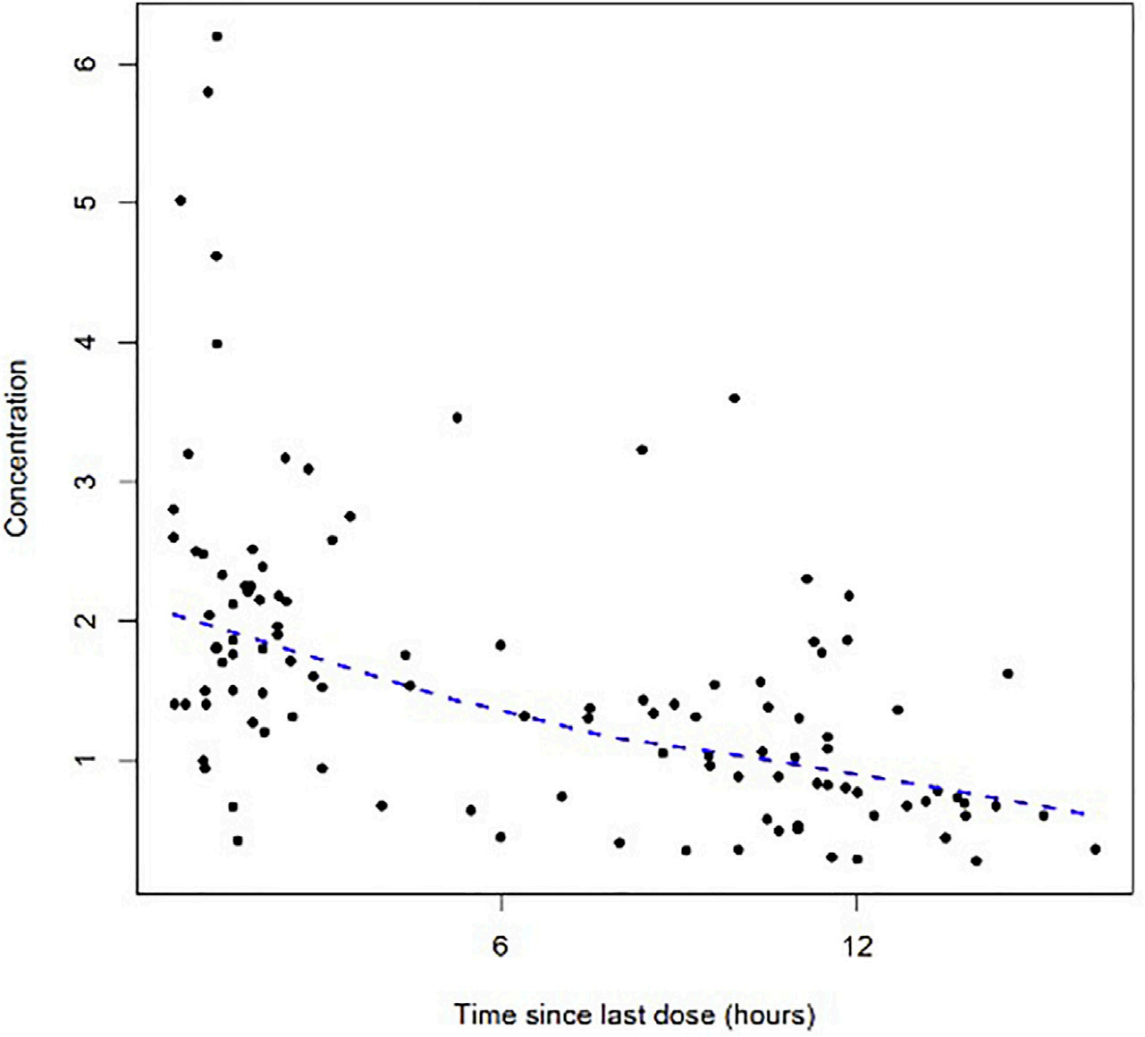
Dose-normalized serum concentration–time profiles of colistin.

A total of 112 colistin concentrations from 42 patients with a range of 0.28–6.20 mg/L were obtained for PK modeling. The colistin concentration versus time after the last dose profile is shown in Figure 1.

The PK characteristic of colistin was well illustrated by a one-compartmental model with linear elimination, which showed a better fit of the observed concentration–time data based on reduction in OFV and residual variability compared to the two-compartmental model. BSV was successfully estimated on both CL volumes in the base model. The proportional error model was selected to evaluate the residual variability. Parameter estimates and diagnostic plots from the base model are provided in Supplementary Table S1, Figure S1.

Covariate model building identified CrCL as a covariate on colistin CL. The final PopPK model is represented as follows:

$$CL\left(L/h\right)=0.994+0.525\times \frac{CrCL}{66.47}$$ (1)

V(L)=20.7 (2)

where CL is the individual clearance, V is the individual volume of distribution, and CrCL is the estimated creatinine clearance. The parameter estimates of the final model are displayed in Table 3.

**TABLE 3:** Population pharmacokinetic parameter estimates from the final model.

Parameter	Estimate	RSE (%)	Shrinkage (%)
Fixed effects			
TVCL (L/h)	0.994	16	
CrCL on CL (θ_1_)	0.525	22	
TVV (L)	20.7	10	
Between-subject variability (BSV^a^)			
BSV_CL [%CV]	30.40%	15	9
Residual variability (RV)			
Proportional error [%CV]	25.10%	16	14

a BSV calculated as $\sqrt{e^{\omega^{2}}-1}$

Abbreviations: TVCL, typical value of clearance; TVV, typical value of volume; CrCL, estimated creatinine clearance.

**FIGURE 2:**
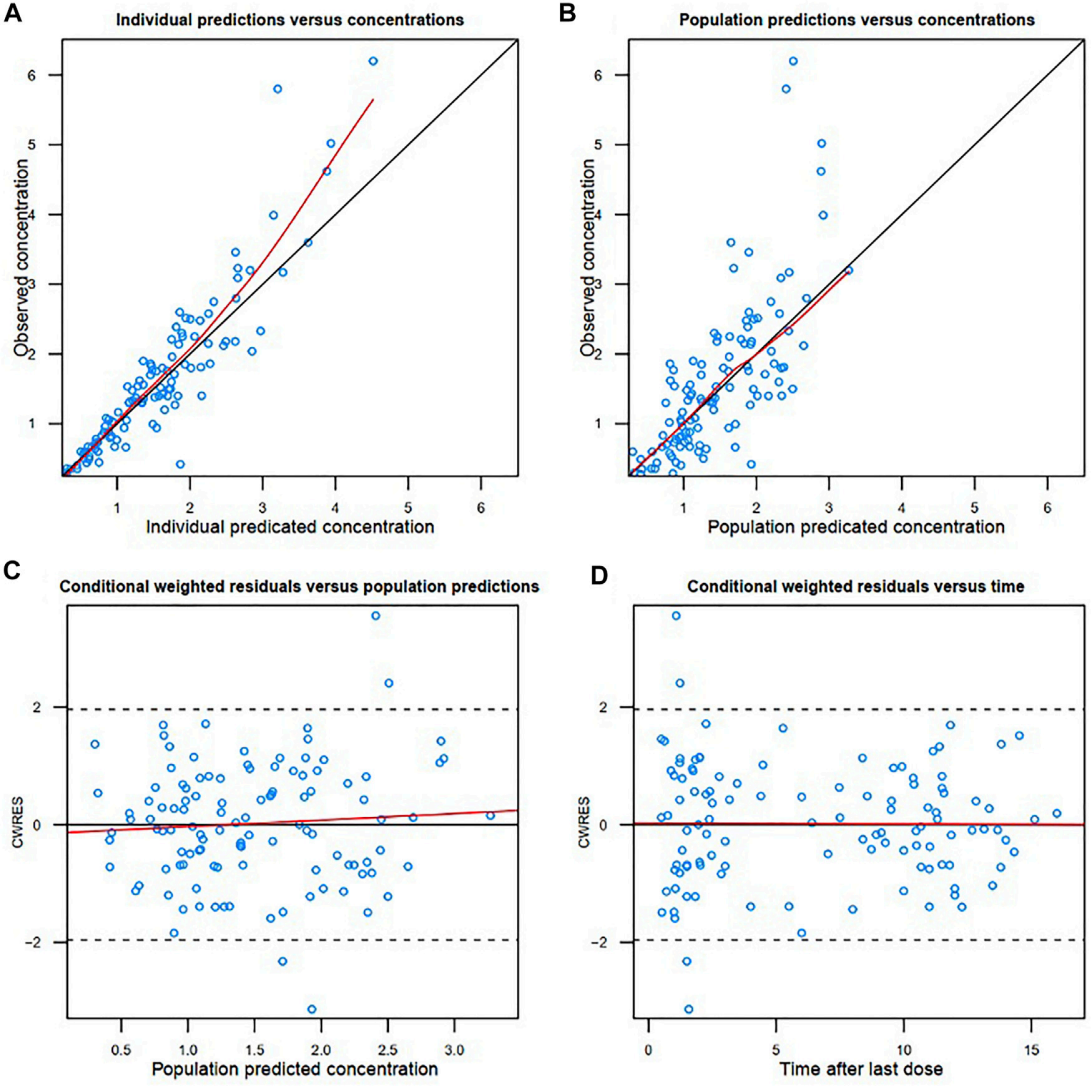
Diagnostic goodness-of-fit plots of the final model. **(A)** Observed concentration (DV) vs. individual predicted concentration (IPRED); **(B)** DV vs. population predicted concentration (PRED); **(C)** conditional weighted residuals (CWRES) vs. PRED; and **(D)** CWRES vs. time. The red lines in the upper panel represent loess smooth lines and linear fit lines, respectively.

The diagnostic goodness-of-fit plots of the final model are shown in Figure 2. The conditional weighted residuals vs. population prediction of the final model showed a stochastic distribution around zero, and most residuals were within an acceptable range (-2 to 2). The median with 95% CI parameter estimates obtained from a 1000-run bootstrap analysis is shown in Supplementary Table S2. The parameter estimates of the final PK model lay within the 95% CI parameter estimates from the nonparametric bootstrap procedure, and the biases between the final model estimates and bootstrapped median parameter estimates were < ±10%, which demonstrated the good stability of the final model. The prediction- and variability-corrected visual predictive check of concentrations versus time after the last dose reflected a good fit between the observations and simulations (Supplementary Figure S2). Overall, the final model provided an adequate description of the data and a good prediction of individual PK parameters of colistin.

Mean ± SD individual empirical Bayesian estimate of CL was 1.74 ± 0.61 L/h across all patients with V estimated at 20.6 L in the population. Interestingly, the PK parameters of colistin sulfate administered intravenously were similar to those of polymyxin B sulfate as we published previously (Yu et al., 2021) (Table 4).

**TABLE 4:** Comparison of pharmacokinetic parameters of colistin sulfate with polymyxin B sulfate.

Study	Subject characteristic	Data	Structural model	PK formula	PK parameter
Colistin sulfate	Adult critically ill patients	Sparse data from a TDM study (112 concentrations from 42 patients)	One-compartment with first-order elimination	CL = 0.994 + 0.525×CrCL/66.47 L/h; V = 20.7 L	CL (L/h): 1.74 ± 0.61; V (L): 20.7
Polymyxin B sulfate (Yu et al., 2021)	Adult critically ill patients	Sparse data from a TDM study (112 concentrations from 32 patients)	One-compartment with first-order elimination	CL = 1.59+(CrCL/80)0.408 L/h; V = 20.5 L	CL (L/h): 1.75 ± 0.43; V (L): 20.5

### Monte Carlo Simulations

**FIGURE 3:**
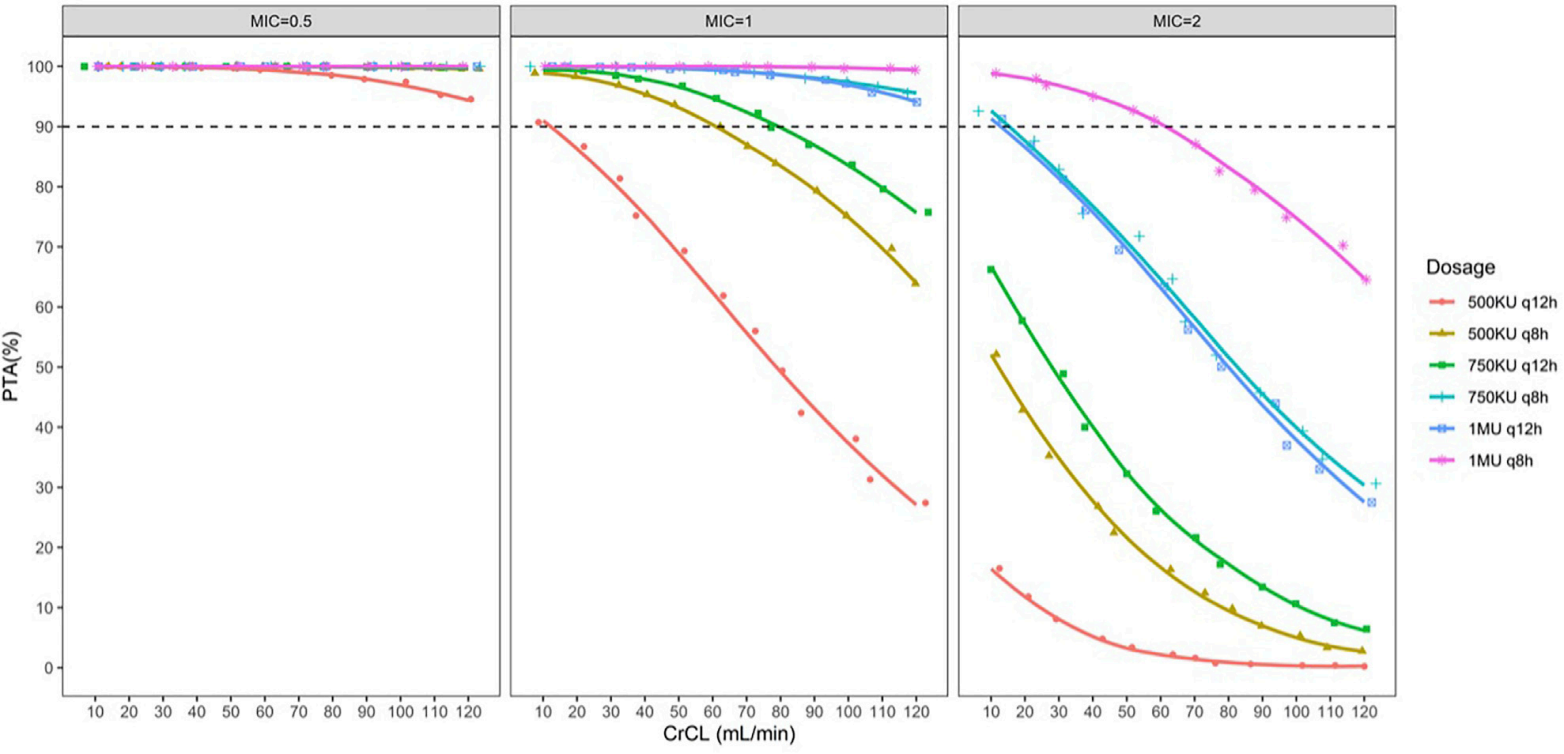
Simulated probability of achieving target attainment (fAUC/MIC ≥15) of colistin sulfate at each MIC (μg/ml) for different dose regimens in patients with various CrCL volumes.

The PTA for the different dose regimens of colistin sulfate at each MIC is shown in Figure 3. The PTA for dose regimens of 500 KU q12h, 500 KU q8h, or 750 KU q12 h was >90% for MICs ≤0.5 μg/ml in patients with various CrCL levels. However, 500 KU q12 h showed subtherapeutic exposure for pathogens with MIC = 1 μg/ml, while 750 KU q12 h could not attain the PTA ≥90% for MICs ≥1 μg/ml in patients with CRCL >80 ml/min. For MIC = 1 μg/ml, 1 MU q12 h or 750 KU q8h could achieve the PTA ≥90% in patients with various CrCL levels. Moreover, all the simulated dose regimens could not achieve the PTA ≥90% for MIC≥ 2 μg/ml. In addition, with the same daily dose, the dosing interval of 12 h had higher PTA achievement than the dosing interval of 8 h.

## Discussion

Polymyxins play an important role in the treatment of life-threatening infections caused by CRO. CMS is the only available form of colistin for parenteral use in most countries, which is administered as an inactive prodrug that undergoes slow conversion to colistin. However, there is a parenteral product of colistin sulfate available in China, but the clinical data on colistin sulfate are very limited. To optimize the use of colistin sulfate in critically ill patients, it is essential for clinicians to understand its pharmacokinetics and clinical outcomes.

In the current study, the result of the pharmacokinetic modeling showed that a one-compartmental model with first-order elimination along with CrCL as a covariate on clearance was optimally fit. The mean ± SD individual empirical Bayesian estimate of CL of colistin was 1.74 ± 0.61 L/h, which is close to the CL of polymyxin B (1.75 ± 0.43) estimated in the study we previously reported (Yu et al., 2021) and another study investigated in Chinese adult patients (median CL: 1.78 L/h) (Wang et al., 2020), whereas it was lower than the one reported in non-Chinese patients (Miglis et al., 2018; Manchandani et al., 2018). Structurally, the major difference between colistin and polymyxin B is the position of sixth amino acid which is the phenylalanine residue in polymyxin B, whereas colistin possesses the leucine residue. Therefore, colistin sulfate and polymyxin B sulfate showed almost the same pharmacokinetic characteristics as they have a very similar molecular structure (Table 4). In contrast, CMS is administered parenterally in the form of the inactive prodrug and undergoes slow conversion to the active form (colistin). Thus, the pharmacokinetic profiles of colistin for intravenous administration of CMS are totally different from our study (Zabidi et al., 2021). As we discussed in our previous study on polymyxin B (Yu et al., 2021), although several studies indicated that polymyxin B cannot be remarkably eliminated from kidneys and its clearance is independent of CrCL (Zavascki et al., 2008; Kwa et al., 2011; Sandri et al., 2013; Thamlikitkul et al., 2016), the data from recent studies performed on Chinese patients showed a contradictory result that CrCL was a statistically significant covariate influencing polymyxin B clearance (Wang et al., 2020; Li et al., 2021; Wang et al., 2021; Yu et al., 2021). Wang et al. (2020) and Yu et al. (2021) defined CrCL as a significant impacting factor on polymyxin B clearance in critically ill patients, while Li et al. (2021) also verified it in renal transplant patients, which also suggested that dosing adjustment should be based on renal function. In our study, CrCL was a significant covariate on the clearance of colistin, which is similar to previous findings in the polymyxin B PK study in Chinese patients, indicating patients with renal insufficiency had higher exposure to colistin sulfate.

It should be noted that the currently used colistin sulfate in China was reapproved for clinical use by the Chinese National Medical Products Administration (NPMA) in 2018. Thus, modern pharmacological data including the clinical efficacy and side effects have not been reported. In our study, we enrolled 42 CRO-infected patients, of which 25 (59.52%) patients achieved clinical anti-infective success after colistin sulfate treatment. The clinical efficacy of colistin sulfate in the current study was similar to the efficacy of polymyxin B reported by Lu et al. (2021), who performed a retrospective study on Chinese patients in which 57.6% of patients with CRO infections achieved clinical success after treating with polymyxin B. However, the daily dose of colistin sulfate and polymyxin B was different in these two studies. The median daily dose of polymyxin B in the study by Lu et al. (2021) was 1.72 mg/kg , while the median daily dose of colistin sulfate was 1.04 mg/kg. Accordingly, despite the significantly lower daily dose used, colistin sulfate attained similar clinical efficacy compared with polymyxin B sulfate. We estimated the AUC_0-24_ at steady state (AUC_0-24,ss_) of colistin sulfate for each patient via the maximum *posteriori* probability (MAP) and the Bayesian function using the final PK model as the Bayesian prior. The mean ± SD of AUC_0-24,ss_ was 39.39 ± 14.47 mgh/L, which was below the clinical PK/PD target of polymyxin B sulfate (AUC_0-24,ss_ ≥ 50 mgh/L) recommended by the international consensus guidelines for the optimal use of the polymyxins published in 2019 (Tsuji et al., 2019). Therefore, the clinical PK/PD target might be different between colistin sulfate and polymyxin B sulfate. The possible reason for the different clinical PK/PD targets might be their difference in antimicrobial activity. An *in vitro* study which compared the potency between colistin and polymyxin B showed that colistin exhibited slightly greater potency than polymyxin B against isolates with lower MIC values (≤2 μg/ml) for both compounds (Sader et al., 2015). Taken together, the clinical PK/PD target of colistin sulfate might be lower than that of polymyxin B sulfate.

*In vitro* and animal studies pointed out that *f*AUC/MIC is the PK/PD index that is best correlated with the efficacy of colistin (Tsuji et al., 2019). The most recent study of systemically administered colistin sulfate against *Acinetobacter baumannii* and *Pseudomonas aeruginosa* in murine thigh models was used to determine *f*AUC/MIC target for various magnitudes of bacterial kill (Cheah et al., 2015). The *f*AUC/MIC values to obtain a 1-log_10_ reduction in bacterial count for experimental thigh infection ranged from 6.6–10.9 for *Pseudomonas aeruginosa* and from 3.5–13.9 for *Acinetobacter baumannii* (Cheah et al., 2015). Therefore, fAUC/MIC ≥15 was determined as the PK/PD target in our Monte Carlo simulations. The plasma unbound fraction of colistin is about 0.5 for critically ill patients (Cheah et al., 2015).

It should be noted that this PK/PD target did not involve the efficacy of colistin sulfate for Enterobacteriaceae. Subsequently, several dose regimens were simulated in virtual patients to identify the suitable dose regimen of colistin sulfate according to the PTA achievement. The recommended dose regimens according to the label sheet of colistin sulfate are 500 KU q12h, 500 KU q8h, or 750 KU q12 h that could attain PTA >90% for MICs ≤0.5 μg/ml. However, 500 KU q12 h was of suboptimal exposure for MIC ≥1 μg/ml. Furthermore, though 500 KU q8h or 750 KU q12 h could attain PTA >90% for MIC = 1 μg/ml, in part of patients with renal insufficiency, it could pose a high risk of suboptimal exposure for patients with CrCL >80 ml/min, whereby 1 MU q12 h was recommended. However, all the simulated dose regimens could not achieve the PTA ≥90% for MIC≥ 2 μg/ml. It is important to note that the PK/PD target of colistin sulfate was derived from a study in which colistin sulfate was used as monotherapy. However, in clinical practice, to prevent the heteroresistance of polymyxins, polymyxins are often used in combination with one or more additional agents (e.g., carbapenem and tigecycline). The combination of carbapenem with polymyxin against Gram-negative bacteria is of high synergy rates *in vitro* (Zusman et al., 2013).

Nephrotoxicity is the main toxicity of concern with the treatment of polymyxins. However, it is amazing that no patient developed colistin sulfate-related nephrotoxicity in our study. Our previously published data showed that C_min_ should be maintained below 3.13 mg/L to prevent polymyxin B-related nephrotoxicity (Han et al., 2022). Considering that polymyxin B and colistin have very similar molecular structures and PK characteristics, it is reasonable to conclude that the safety threshold of nephrotoxicity of colistin sulfate approaches that of polymyxin B. In the current study, most patients had a C_min_ below 3.13 mg/L. In addition, as we discussed earlier, 1 MU q12 h was recommended for MIC = 1 μg/ml in patients with CrCL >80 ml/min, and the C_min_ of this dose regimen in simulated virtual patients was 2.33 ± 1.13, which was also below the threshold of nephrotoxicity. Therefore, despite higher exposure to colistin in patients with renal insufficiency, dose reduction was not recommended as the exposure was still below the threshold of nephrotoxicity. In addition, patients with severe infections frequently suffer from acute kidney injury, which can be soon reversed when the infections are alleviated, whereby the reduction of dose at acute kidney injury occasions may lead to subtherapeutic exposure when the AKI are alleviated.

The retrospective nature of the study imposes some limitations. First, this study enrolled a relatively small number of patients, leading to a lack of external validation of the PK model. Second, biases could not be controlled completely in evaluating the clinical outcomes of colistin sulfate as this was a retrospective and small study cohort. Third, as the PK/PD target for simulations was derived from a pre-clinical study, the PTA endpoints of dose regimens should be further confirmed clinically. Fourth, it was a limitation that we used retrospective data to perform the Naranjo test to define whether AKI was caused by colistin sulfate or not in those two patients who developed AKI during the colistin sulfate treatment. Finally, future prospective studies should evaluate the PK of colistin sulfate and its clinical outcomes with a larger population.

In general, the present study is the first study that investigated the clinical pharmacokinetics of colistin sulfate administered intravenously in which renal function significantly affects the PK of colistin. In addition, the currently recommended dose regimen of colistin sulfate, according to the label sheet, could pose a risk of suboptimal exposure for MIC ≥2 μg/ml; the off-label dose of 1 MU q12 h might be the alternative choice for MIC = 1 μg/ml in patients with CrCL >80 ml/min. Moreover, despite higher exposure to colistin in patients with renal insufficiency, dose reduction was not recommended as the current dose regimens were still below the threshold of nephrotoxicity and considered safe.

## Data Availability

The original contributions presented in the study are included in the article/Supplementary Material; further inquiries can be directed to the corresponding authors.
