# Supplementary material for: Population Pharmacokinetics of Colistin Sulfate in Critically Ill Patients: Exposure and Clinical Efficacy
Source: Front Pharmacol. 2022 Jun 16;13:915958. doi: 10.3389/fphar.2022.915958 (PMC9243584; doi:10.3389/fphar.2022.915958)
Supplement: Supplementary file 1 [file DataSheet1.PDF]

Table S1 Population pharmacokinetic parameter estimates from the base model

Parameter	Estimate	RSE	shrinkage
**Fixed Effects**			
TVCL[L/h]	1.63	7%	
TVV[L]	20.6	10%	
**Between-subject Variability (BSV[a])**			
BSV_CL [%CV]	38.70%	14%	6%
**Residual Variability (RV)**			
Proportional Error [%CV]	26%	16%	15%

[a]BSV calculated as $\sqrt{e^{\omega^2}-1}$

Table S2 Linezolid PopPK parameter estimates from the final model and bootstrap results

Parameter	Final model		shrinkage			Bias (%)
	Estimate	RSE(%)	2.5th percentile	Median Estimate	97.5th percentile	
θ_{CL}[L/h]	0.994	16	0.67	0.98	1.32	-1.34
θ_{V}[L]	20.7	10	16.45	20.91	24.85	1.00
CrCL on CL (θ_1)	0.525	22	0.28	0.53	0.77	1.20
Inter-individual variability						
ω_{CL} [%]	30.4	30	19.24	29.52	38.39	-2.91
Residual Variability						
σ_{pro}(%)	25.1	16	20.82	24.85	28.74	-0.99

PopPK, population pharmacokinetic; RSE(%), relative standard error; θ_{CL}, typical value of apparent clearance; θ_V, typical value of apparent volume of distribution; θ_1, allometric value for CrCL as covariate for CL; ω_{CL}, square root of inter-individual variance for CL; σ_{pro}, residual variability for proportional error.

Bias (%)=(Median Estimate $_{Bootstrap}$ - Estimate $_{Final\ model}$)/Estimate $_{Final\ model}$ × 100%

Figure S1

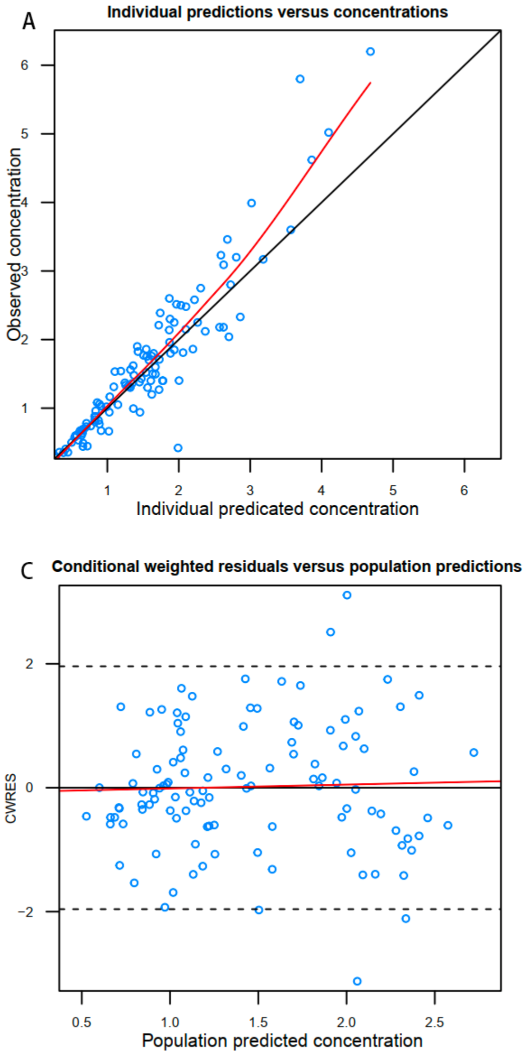

Figure S1. Diagnostic goodness-of-fit plots of the base model. (A) Observed concentration (DV) vs. individual predicted concentration (IPRED); (B) DV vs. population predicted concentration (PRED); (C) conditional weighted residuals (CWRES) vs. PRED; and (D) CWRES vs. time. The red lines in the upper panel represent loess smooth lines and linear fit lines, respectively.

Figure S2

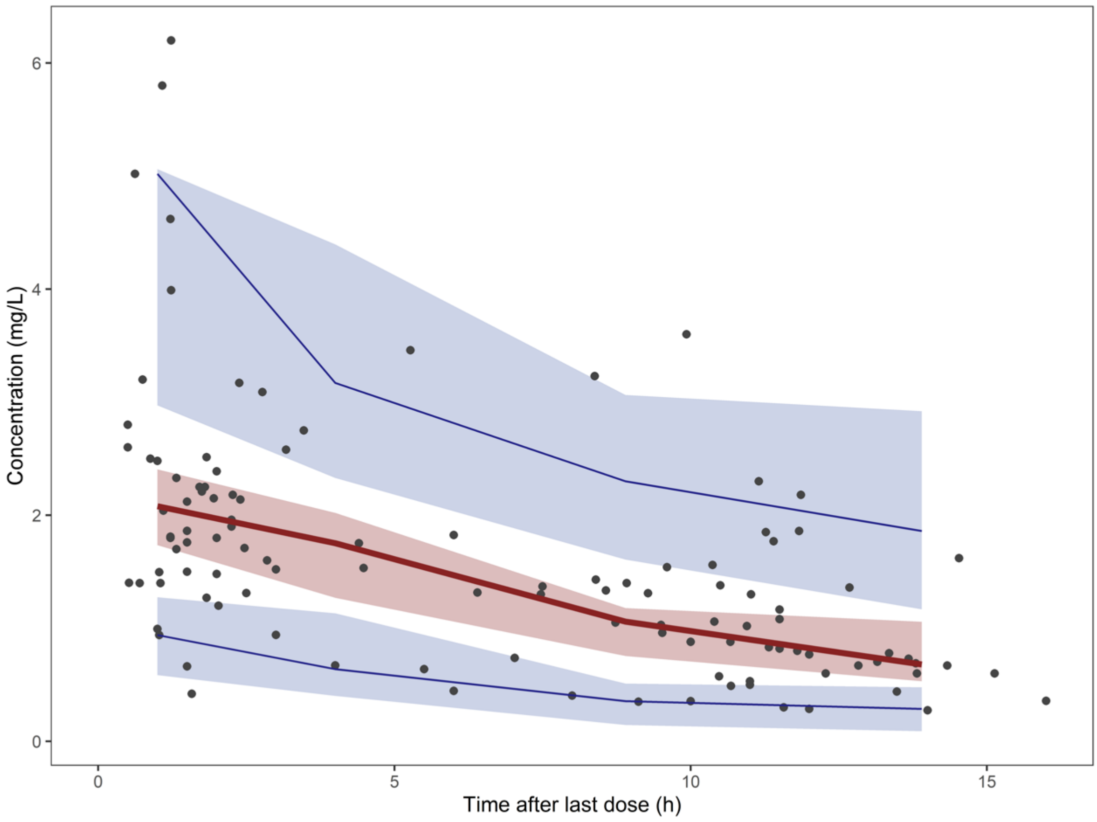

Figure S2. Prediction- and variability-corrected visual predictive check (pvcVPC) plot of the final model. The red solid lines represent the median observed concentration, and the semitransparent red fields represent the simulation-based 95% confidence intervals (CIs) for the median. The observed 5th and 95th percentiles are represented by red dashed lines, and the 90% CIs for the corresponding model predicted percentiles are shown as semitransparent blue fields. The observed concentrations are represented by dark dots.
